# Cord Blood RSV-Neutralizing Antibodies and Risk of Hospitalization for RSV-Associated Acute Respiratory Infection in Vietnamese Children: A Case–Cohort Study

**DOI:** 10.3390/vaccines13090963

**Published:** 2025-09-11

**Authors:** Michiko Toizumi, Yutaro Yamagata, Hien Anh Thi Nguyen, Hirono Otomaru, Hoang Huy Le, Hiroyuki Moriuchi, Jean-Francois Eleouet, Marie-Anne Rameix-Welti, Makoto Takeda, Hung Thai Do, Lay-Myint Yoshida

**Affiliations:** 1 Department of Pediatric Infectious Diseases, Institute of Tropical Medicine, Nagasaki University, Nagasaki 852-8523, Japan; y_yutaro@nagasaki-u.ac.jp (Y.Y.); otomaru-h@nagasaki-u.ac.jp (H.O.); hungdt02@yahoo.com (H.T.D.); 2 National Institute of Hygiene and Epidemiology, Hanoi 100000, Vietnam; hienanh75@yahoo.com (H.A.T.N.); lehuyhoang2010@gmail.com (H.H.L.); 3 National Research Center for the Control and Prevention of Infectious Diseases (CCPID), Nagasaki University, Nagasaki 852-8523, Japan; hiromori@nagasaki-u.ac.jp; 4 Unité de Virologie et Immunologie Moléculaires (VIM), Institut National de Recherche pour l’Agriculture, l’Alimentation et l’Environnement (INRAE), Université Paris-Saclay, 78350 Jouy-en-Josas, France; jean-francois.eleouet@inrae.fr; 5 Molecular Mechanisms of Multiplication of Pneumoviruses Unit (M3P), Institut Pasteur, Université Paris-Saclay, Université de Versailles St. Quentin, Université Paris Cité, UMR 1173 (2I), INSERM, Centre National de Reference Virus des Infections Respiratoires (CNR VIR), Assistance Publique des Hôpitaux de Paris, 75015 Paris, France; marie-anne.rameix-welti@pasteur.fr; 6 Faculty of Medicine, Graduate School of Medicine, The University of Tokyo, Tokyo 113-0033, Japan; mtakeda@m.u-tokyo.ac.jp; 7 Pasteur Institute in Nha Trang, Nha Trang 650000, Vietnam

**Keywords:** respiratory syncytial virus, neutralizing antibodies, cord blood, maternal antibodies, infants, maternal immunization, case–cohort study

## Abstract

Background: Respiratory syncytial virus (RSV) is a leading cause of lower respiratory tract infections in children, particularly severe during infancy. Maternal RSV-specific neutralizing antibodies (nAbs), transferred via the placenta, may provide protection in early infancy, but the extent and duration of protection remain uncertain. Objective: We investigated the association between cord blood RSV-A nAb levels and the risk of hospitalization due to RSV-associated acute respiratory infection (RSV-ARI) by 24 months of age. Methods: We conducted a case–cohort study nested within a birth cohort in Nha Trang, Vietnam. From the full cohort (*n* = 1977), a random subcohort of 392 infants and all 66 infants hospitalized for RSV-ARI by age 24 months were included for RSV-A nAb testing. RSV-A nAb titers at birth were categorized into three groups in the subcohort (low: lowest quartile; middle; interquartile; high: highest quartile). Weighted Cox proportional hazards regression was used to estimate hazard ratios (HRs) for RSV-ARI hospitalization. Results: The incidence of RSV-ARI hospitalization was 17.92 per 1000 person-years by 24 months, and 25.40 per 1000 person-years among infants aged <12 months. Among infants aged <6 months, those in the low nAb group had a significantly higher risk of hospitalization compared to the middle nAb group (adjusted HR: 4.05; 95% CI: 1.51–10.89). Maternal anemia was consistently associated with increased risk. Conclusions: Lower RSV-nAb titers at birth were associated with an increased risk of RSV-ARI hospitalization during early infancy. These findings support the importance of maternal immunization strategies to enhance infant protection against RSV.

## 1. Introduction

Respiratory syncytial virus (RSV) is the leading cause of acute lower respiratory tract infections (ALRI) in infants, particularly during the first few months of life. Globally, RSV accounts for more than 3 million hospitalizations and approximately 118,000 deaths annually among children under five years of age, the majority of which occur in low- and middle-income countries (LMICs) [1]. In Vietnam, RSV is consistently identified as a major pathogen among hospitalized infants with respiratory illnesses [2].

Maternal immunization [3] and long-acting monoclonal antibodies such as nirsevimab [4] represent promising approaches for protecting young infants from severe RSV illness. The protective effect of maternally derived RSV-neutralizing antibodies transferred through the placenta is a key focus in this context. Although these antibodies are believed to provide temporary protection in early infancy, existing studies have yielded mixed findings regarding the association between antibody titers and risk of RSV disease. Several studies have suggested a protective role for maternal antibodies, including early observations from the US and UK [5,6,7], a study from rural Mozambique [8], and a Danish study that found a temporal association between average maternal antibody levels and seasonal trends of RSV hospitalization [9]. Similarly, a recent study in Mali [10] found that higher cord blood RSV-neutralizing antibody (nAb) titers were associated with a reduced risk of RSV illness in infants. In contrast, other studies in various settings, including Arizona [11], Alaska [12], rural Nepal [13], and coastal Kenya [14], did not demonstrate a clear correlation between individual antibody levels and protection against RSV infections or severe diseases. Differences in antibody assays, exposure timing, viral epidemiology, study designs, and study settings may account for this variability.

In this study, we aimed to estimate the incidence of RSV-associated acute respiratory infection (RSV-ARI) hospitalizations during the first two years of life in a birth cohort in Nha Trang, Vietnam. We further examined whether RSV-nAb titers in cord blood were associated with subsequent hospitalization for RSV-ARIs and explored the maternal and perinatal factors related to RSV-ARI hospitalization risk in early childhood.

## 2. Materials and Methods

### 2.1. Study Design and Setting

We conducted a case–cohort study nested within a prospective birth cohort established at Khanh Hoa General Hospital (KHGH), a provincial hospital in Nha Trang City, Vietnam. The cohort enrolled women residing in 16 communes within the hospital’s catchment area who delivered singleton live births at KHGH between July 2017 and September 2018 [15]. Each child was followed from birth until hospitalization for respiratory syncytial virus-associated acute respiratory infection (RSV-ARI), relocation out of the catchment area, death, or reaching 24 months of age, whichever occurred first.

### 2.2. Participants

Children with available cord blood samples were eligible for the analysis. A random subcohort comprising 20% of the eligible children was selected as the reference group. All RSV-ARI hospitalized cases within the full cohort were included regardless of the subcohort status.

### 2.3. RSV-ARI Hospitalization Surveillance and Case Definition

Hospitalizations were identified through an ongoing pediatric ARI surveillance system [16]. Birth cohort children admitted to KHGH with cough and/or difficulty breathing [17] were enrolled in the surveillance. RSV-ARI hospitalization was defined as hospitalization meeting this symptom criterion, with RSV detected using reverse transcription polymerase chain reaction (RT-PCR) in nasopharyngeal swabs collected at admission. Admission decisions were made by the treating clinicians; no local guideline mandated admission or RSV testing by age group.

### 2.4. Data Collection

At delivery, demographic and perinatal information were collected, and cord blood samples were obtained. Clinical data were recorded during hospitalization for ARIs and nasopharyngeal swabs were collected for microbiological testing [16].

### 2.5. Virological Testing

Viral nucleic acids were extracted from nasopharyngeal swab specimens using the QIAamp Viral RNA Mini Kit (QIAGEN Inc., Valencia, CA, USA). An established in-house multiplex (RT-)PCR assay [16] was used to screen for 13 common respiratory viruses, including RSV, influenza viruses, parainfluenza viruses, human metapneumovirus, adenovirus, bocavirus, and enteroviruses. All virus-positive samples were confirmed by hemi-nested (RT-)PCR.

For RSV-positive samples, the second variable region of the G gene was amplified by RT-PCR using previously described methods to distinguish between RSV-A and RSV-B [18]. Amplicons were purified with ExoSAP-IT Express (Thermo Fisher Scientific, Waltham, MA, USA) and sequenced using BigDye Terminator v1.1 Cycle Sequencing Kits (Applied Biosystems, Foster City, CA, USA). Sequence analysis was performed using 3130xl Genetic Analyzer (Applied Biosystems). Subgroup classification was based on the obtained sequence information.

### 2.6. Bacteriological Testing

For bacterial identification, nasopharyngeal swab specimens were cultured using standard microbiological methods [19]. No PCR testing was conducted for bacterial pathogens.

### 2.7. Neutralizing Antibody Testing

Cord blood plasma was tested for neutralizing activity against RSV-A using a fluorescence-based neutralization assay. This assay employed a recombinant virus derived from the RSV-A Long strain that expresses mCherry as a reporter [20]. The 50% inhibitory concentration (IC_50_) was calculated and log_2_-transformed for analysis.

### 2.8. Statistical Analysis

The primary analysis estimated the association between cord blood RSV-A nAb titers and the risk of hospitalization for RSV-associated acute respiratory infection (RSV-ARI) using weighted Cox proportional hazards regression models. Follow-up began at birth and ended at the time of RSV-ARI hospitalization or censoring, as defined in the cohort protocol.

RSV-nAb titers were categorized into three groups based on their distribution in the subcohort: the lowest quartile (low), interquartile range (middle 50%), and highest quartile (high). This data-driven classification was used to explore the nonlinear associations between antibody levels and disease risk. To account for the case–cohort design, inverse probability weights were applied to the subcohort, and robust standard errors were clustered by participant ID.

**Figure 1:**
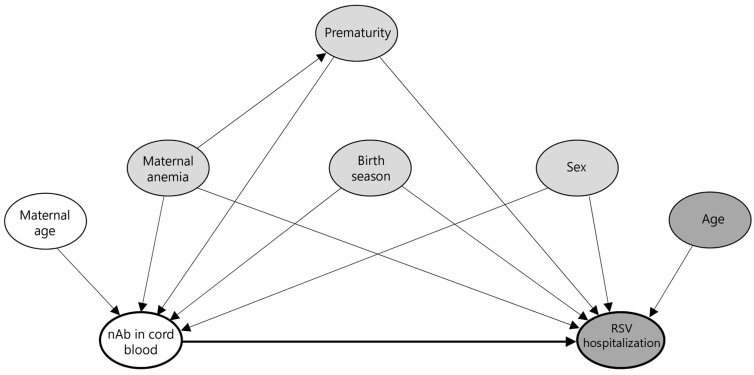
Directed acyclic graph (DAG) illustrating hypothesized causal pathways from cord blood neutralizing antibodies to RSV hospitalization. This DAG represents the assumed causal structure for assessing the association between cord blood neutralizing antibody (nAb) levels and RSV-associated hospitalization. The thick black arrow indicates the primary hypothesized causal pathway from exposure (nAb) to the outcome (RSV hospitalization). Node colors indicate variable roles: white for ancestors of exposure only, dark gray for ancestors of outcome only, and gray for ancestors of both (i.e., potential confounders, including maternal anemia, prematurity, birth season, and sex).

The follow-up time was modeled using predefined age bands (0–5, 6–11, 12–17, and 18–23 months) and treated as a time-dependent variable. Multivariable models were adjusted for sex, prematurity (gestational age < 37 weeks), maternal anemia at delivery (hemoglobin level < 13 g/dL), and birth season (categorized as January–March, April–June, July–September, and October–December). Covariates were selected based on prior knowledge and a hypothesized causal structure represented by a directed acyclic graph (DAG), as shown in Figure 1.

As most RSV-ARI hospitalizations occurred during infancy, we conducted a pre-specified additional analysis restricted to events occurring before 12 months of age. The interaction terms between the antibody group and age band or maternal anemia were also tested. Sensitivity analyses included low birth weight as a covariate and repeated the analysis after excluding low-birth-weight infants.

Weighted incidence rates of RSV-ARI hospitalization were computed as weighted events divided by weighted person-years and reported per 1000 person-years. Case–cohort sampling weights were applied to both the event counts and the person-time. Person-time accrued from birth to the event or censoring. For the age-restricted estimates (0–12 and 0–6 months), follow-up was truncated at 12 or 6 months for all participants, and only events within the respective window were counted. Because complete follow-up (out-migration and deaths through 24 months) was available only for the subcohort and RSV-ARI hospitalized cases, all-cause ARI hospitalization incidence for the full cohort was not estimated.

## 3. Results

### 3.1. Study Population

**Figure 2:**
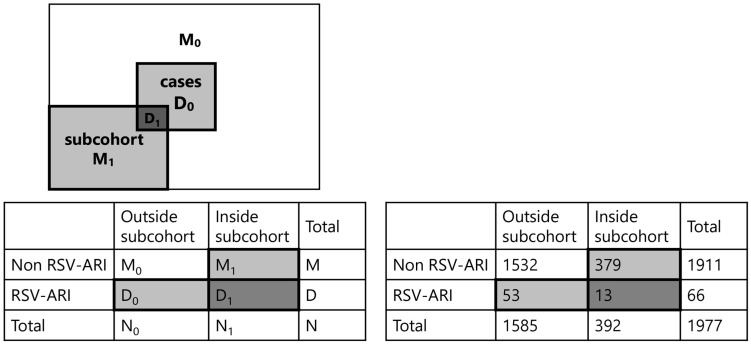
Overview of the case–cohort design and its application in this study. Left panel: Conceptual diagram of the case–cohort design. From the full cohort, a subcohort was randomly selected at baseline, which included both cases (D_1_) and non-cases (M_1_). All additional cases occurring outside the subcohort (D_0_) were also included. The analysis compares all cases (D_0_ + D_1_) with the subcohort (D_1_ + M_1_). Right panel: Implementation of case–cohort design in the present study. Among the 1977 children in the full birth cohort, 66 were hospitalized for RSV-associated acute respiratory infection (RSV-ARI). A 20% subcohort of 392 children was randomly sampled from the entire cohort. All RSV-ARI cases—both those within the subcohort (D_1_) and outside (D_0_)—were included. Analysis was conducted by comparing all cases (D_0_ + D_1_) to the subcohort (D_1_ + M_1_).

Of the 2015 children enrolled in the birth cohort, 1977 (98.1%) had cord blood samples available for RSV neutralization testing. A total of 66 children were hospitalized for RSV-ARI by 24 months of age, including 24 with RSV-A, 31 with RSV-B, and 11 with untyped RSV. A subcohort of 392 children was randomly selected, of whom 13 later developed RSV-ARI hospitalization (Figure 2).

### 3.2. Incidence of RSV-ARI Hospitalization

The weighted incidence rate of RSV-ARI hospitalization was 17.92 per 1000 person-years over the 24-month follow-up period. Within the first 12 months of life, the weighted incidence rate was substantially higher at 25.40 per 1000 person-years. It was 26.09 per 1000 person-years in the first six months of life.

### 3.3. Cord Blood Antibodies and RSV-ARI Hospitalization Risk (Primary Analysis)

**Figure 3:**
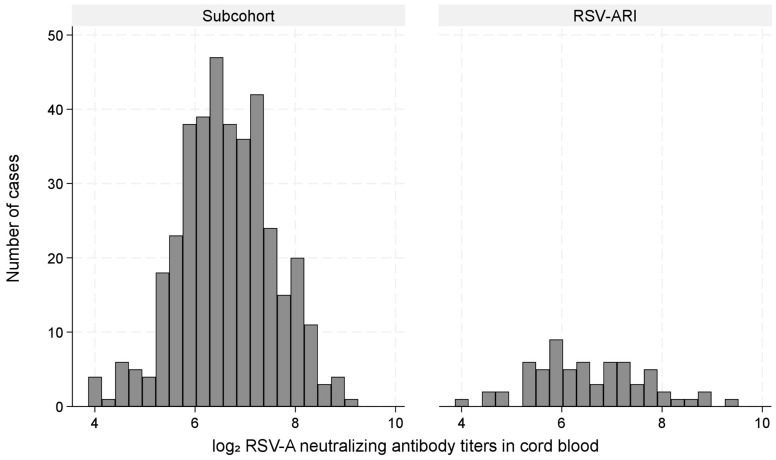
Distribution of log_2_ RSV-A neutralizing antibody titers in cord blood among the subcohort and RSV-ARI hospitalization cases.

Figure 3 shows the distribution of log_2_ RSV-A nAb titers in the subcohort and RSV-ARI hospitalization cases with a substantial overlap. The mean (standard deviation) titers were 8.75 (1.37) in the subcohort and 8.83 (1.61) among cases.

Table 1 presents the weighted Cox regression results for RSV-ARI hospitalizations over 24 months. The analysis included 392 infants in the subcohort and 66 cases. Among the subcohort, 98 (25.0%) had low antibody titers, 196 (50.0%) had middle titers, and 98 (25.0%) had high titers. Among the RSV-ARI hospitalization cases, 23 (34.9%) were in the low-titer group, 26 (39.4%) in the middle, and 17 (25.8%) in the high group. Compared to the subcohort, a higher proportion of RSV-ARI hospitalization cases were in the low-titer group, while the proportions in the middle and high groups were similar. Infants with low antibody titers had a higher risk of RSV-ARI hospitalization than those with middle titers (adjusted hazard ratio (aHR): 1.80; 95% CI: 0.93–3.50), with some evidence supporting the association (*p* = 0.083). There was no strong indication of an increased or decreased risk in the high-titer group (aHR: 1.32; 95% CI: 0.68–2.60). Maternal anemia was strongly associated with RSV-ARI hospitalizations (aHR: 4.10; 95% CI: 2.30–7.28; *p* < 0.001), and female sex was suggestive of a protective effect (aHR: 0.56; 95% CI: 0.32–1.00; *p* = 0.048). Other characteristics such as gestational age, maternal age, and mode of delivery showed no substantial differences between cases and the subcohort.

This figure shows the distribution of log_2_-transformed RSV-A neutralizing antibody titers in cord blood, comparing children in the subcohort with those hospitalized for RSV-associated acute respiratory infection (RSV-ARI).

### 3.4. Additional Analysis: 12-Month Follow-Up

**Figure 4:**
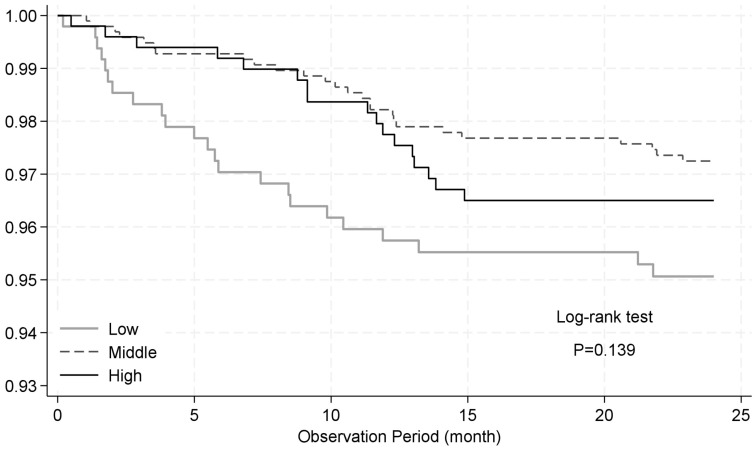
Kaplan–Meier survival curves for time to hospitalization due to RSV-associated acute respiratory infection (RSV-ARI) stratified by antibody titer. Children were stratified into three groups based on cord blood neutralizing antibody titers against RSV-A: lowest quartile (low), interquartile range (middle), and highest quartile (high). The curves show the proportion of children who remained free from RSV-ARI hospitalization until 24 months of age. No statistically significant difference was observed between the groups (log-rank test, *p* = 0.139).

Most RSV-ARI hospitalizations occurred within the first year of life (Figure 4). The 12-month-limited analysis provided stronger evidence of an increased RSV-ARI hospitalization risk associated with low antibody titers (aHR: 2.37; 95% CI: 1.09–5.14; *p* = 0.029), whereas there was little evidence of an association for high titers (aHR: 1.36; 95% CI: 0.61–3.06) (Table 2). Maternal anemia remained strongly associated with RSV-ARI hospitalization (aHR: 5.45; 95% CI: 2.80–10.60), and the suggested protective effect of female sex persisted (aHR: 0.57; 95% CI: 0.29–1.12).

### 3.5. Subgroup, Interaction, and Sensitivity Analyses

We explored the age-specific association between cord blood RSV antibody titers and the risk of RSV-ARI hospitalization by estimating models stratified by age band. Among infants aged 0–5 months, low titers were associated with an increased risk of RSV-ARI hospitalization, with strong evidence supporting this association (aHR: 4.05; 95% CI: 1.51–10.89), while no protective association was observed for high titers (aHR: 1.17; 95% CI: 0.33–4.07). In older age bands, hazard ratio estimates for both low- and high-titer groups became more variable, with wide confidence intervals crossing unity. Notably, in the 18–23-month group, estimates were unstable due to sparse events, particularly in the high-titer group, where the incidence was extremely low (Table 3A). These findings suggest that the protective effect of maternally derived RSV antibodies is most evident during infancy. There was no evidence of an interaction between antibody titer groups and maternal anemia (*p* = 0.53) (Table 3B). Sensitivity analyses excluding children with low birth weight showed similar results, indicating a limited influence from the subcohort overrepresentation of low birth weight (Supplementary Table S1).

### 3.6. RSV-Neutralizing Antibody Titer by Infecting RSV Type

**Figure 5:**
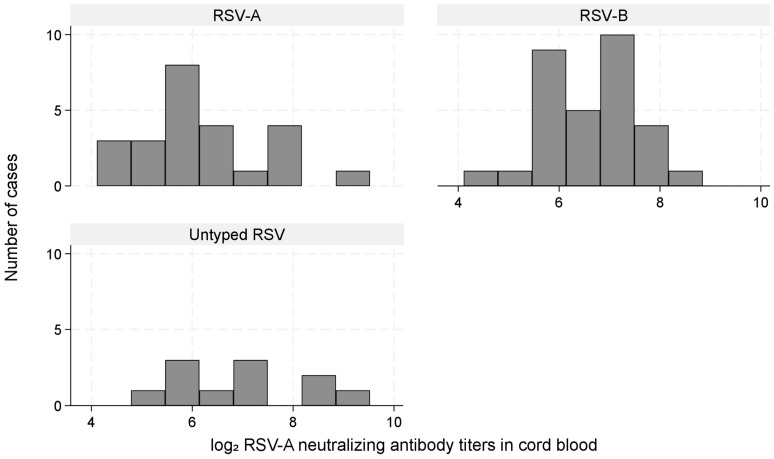
Distribution of log_2_ RSV-A neutralizing antibody titers among RSV-ARI hospitalization cases stratified by RSV type (A, B, untyped). Histograms illustrating the distribution of cord blood RSV-A neutralizing antibody titers (log_2_ scale) among RSV-ARI hospitalization cases stratified by type: RSV-A (*n* = 24), RSV-B (*n* = 31), and untyped (*n* = 11). Differences among the groups were not statistically significant (Kruskal–Wallis test, *p* = 0.20).

The neutralization assay was performed using the RSV-A strain. Of the 66 RSV-ARI hospitalizations, the infecting type was RSV-A in 24 (36%), RSV-B in 31 (47%), and untyped in 11 (17%). Among RSV-ARI hospitalization cases, mean (SD) log_2_ IC_50_ values in the cord blood were 6.29 (1.17) for RSV-A, 6.62 (0.97) for RSV-B, and 6.92 (1.30) for untyped cases (Kruskal–Wallis test, *p* = 0.20; Figure 5). In type-specific analyses, low titers were associated with RSV-A hospitalization (aHR: 2.80; 95% CI: 1.00–7.86), whereas estimates for RSV-B were unstable because of sparse data and quasi-complete separation, particularly because the subcohort included no RSV-B cases in the high-titer group (Table 4).

Hazard ratios were estimated using separate weighted Cox regression models for RSV-A and RSV-B outcomes, adjusting for age band, sex, prematurity, maternal anemia, and birth season. The middle-titer group served as the reference. Robust standard errors were clustered by participant ID.

The RSV-B model yielded unstable estimates due to sparse data and quasi-complete separation. Among the 31 RSV-B cases, only 7 were in the subcohort used for weighted Cox regression, and these were unevenly distributed across titer groups: 6 in the low-titer group, 1 in the middle-titer group, and none in the high-titer group. This imbalance precluded reliable estimation of the hazard ratio for the high-titer group.

### 3.7. Representativeness and Clinical Features

Supplementary Table S2 compares the subcohort with the full cohort. The distributions were similar, except for a slightly higher proportion of low-birth-weight infants in the subcohort. Supplementary Table S3 compares RSV-ARI cases with all hospitalized ARI cases in children aged <2 years, showing younger age and more frequent wheezing and hypoxia among RSV-ARI cases.

## 4. Discussion

This case–cohort study in Nha Trang, Vietnam, assessed the burden of RSV-ARI hospitalization during the first two years of life, examined the association between cord blood RSV-nAb titers and subsequent RSV-ARI hospitalization, and explored maternal and perinatal risk factors. We observed a high burden of RSV-ARI hospitalization, especially during early infancy. Lower cord blood RSV-nAb titers were associated with an increased risk, particularly within the first year of life. Maternal anemia also emerged as a consistent risk factor independent of antibody levels.

### 4.1. High Incidence of RSV-ARI Hospitalization in Infancy

In our study, the incidence of RSV-ARI hospitalization was 26.09 per 1000 person-years among infants aged <6 months and 25.40 per 1000 person-years for those aged <12 months. The incidence declined to 17.92 per 1000 person-years when all children under 24 months of age were considered. These findings highlight the substantial burden of RSV-associated hospitalization in infancy. Our estimates align with global data showing high RSV-associated hospitalization rates in infancy, with our <6-month incidence falling between those reported in Kenya (13.4 per 1000) [21] and South Africa (70 per 1000) [22], and is comparable to the estimate from Singapore (33.5 per 1000) [23]. Incidence varied by epidemic year—for example, in Santa Rosa, Guatemala, it was 8.7 per 1000 in 2008 and 65.2 per 1000 in 2009 [24]. For children aged 1–2 years, the incidence was lower than in younger children, which is also consistent with our observation: 0.7 per 1000 in Quetzaltenango and 1.2–4.8 per 1000 in Santa Rosa, Guatemala (depending on year) [24], 4.4 in Kenya [21], 10 in South Africa [22], and 19.1 in Nicaragua [25]. These age-specific trends reaffirm the critical need for preventive interventions focused on the first year of life, when the risk of RSV-ARI hospitalization is highest.

### 4.2. Cord Blood RSV-nAb Titers and Risk of RSV-ARI Hospitalization

Infants with lower cord blood RSV-nAb titers had a higher risk of RSV-ARI hospitalization during the first 12 months of life, particularly in the first six months. This finding supports prior studies indicating protection conferred by maternally derived antibodies [5,7,9]. However, the association did not follow a strictly dose-dependent pattern; higher titers did not correspond to proportionally greater protection. This observation may be consistent with findings from a recent maternal RSV vaccine trial [26], which showed that absolute anti-F IgG antibody levels were not strongly correlated with protection, whereas the relative fold-increase following vaccination was more predictive. The study from a Malian birth cohort reported that infants with cord blood RSV-A-neutralizing antibody titers above a certain threshold had a markedly reduced risk of RSV-associated illness, although a strictly dose-dependent relationship was not evident [10]. These findings suggest that increases in antibody quantity may not necessarily translate into proportional gains in protection. One possible explanation is that higher antibody titers may reflect repeated maternal exposure to RSV in high-transmission settings. In such environments, both mothers and their infants may face increased exposure risk, which could outweigh the protective effects of maternally derived antibodies. This ecological confounding could partially explain the absence of a dose-dependent association between antibody titers and protection in our findings.

Moreover, our results underscore that maternally derived protection may be most relevant during early infancy and may wane rapidly, contributing to increased vulnerability later in the first year of life. This pattern is consistent with previous studies describing the kinetics of maternally transferred anti-RSV IgG antibodies. For instance, studies from China and Kenya have estimated the half-life of anti-RSV IgG at approximately 1.4 months and 79 days, respectively [27,28]. In Turkey, Hacimustafaoglu et al. observed a steep decline in IgG positivity from 83% at birth to just 2% by 6 months of age [29]. Such waning of maternal antibodies may contribute to the increased risk of RSV infection later in infancy. These findings are in line with our age-stratified results, in which an association between low cord antibody titers and RSV hospitalization was most evident in infants aged 0–5 months, but not observed in subsequent age bands.

In contrast, prior studies from Arizona [11] (endpoint: RSV LRI, not hospitalization; design: birth cohort with nested case–control; assay: binding serology), Alaska [12] (RSV hospitalization; matched case–control; complement-enhanced PRNT), rural Nepal [13] (RSV infection/severity/timing; community birth cohort; microneutralization), and coastal Kenya [14] (severe hospitalized RSV; hospital-based case–control; PRNT, RSV A2) found no clear individual-level association between cord RSV antibodies and protection. Differences in endpoints, study design, laboratory platforms/strains, and epidemiologic context (e.g., community vs. hospital ascertainment, transmission intensity) likely account for the discrepancy between those findings and ours.

### 4.3. Maternal and Perinatal Risk Factors

In addition to antibody levels, maternal anemia was independently associated with an increased risk of RSV-ARI hospitalization. This is supported by previous studies showing that maternal anemia is linked to elevated risk of respiratory morbidity and infections in children [30,31], possibly due to impaired fetal immune development or higher risk of prematurity and low birth weight [32,33,34]. Maternal anemia also can lead to infantile anemia, and infants with iron-deficiency anemia appear more vulnerable to acute bronchiolitis, including RSV disease, according to recent studies [35,36].

Female sex appeared protective, consistent with previous reports of sex differences in susceptibility and immune response to respiratory infections [37,38,39].

Other factors, such as the birth season and prematurity, showed less consistent associations. Prior studies have shown that infants born shortly before or during the RSV season are at increased risk [40,41], and that preterm infants have a substantially higher RSV-related hospitalization risk [42,43]. Although not definitive in our data, these associations merit further exploration in larger samples.

### 4.4. Strengths

This study has several strengths. First, the use of a population-based birth cohort with active hospital surveillance enabled robust estimation of age-specific incidence. Second, RSV-ARI hospitalization cases were confirmed using standardized RT-PCR testing to minimize outcome misclassification. Third, antibody titers were measured using a standardized microneutralization assay based on recombinant RSV expressing a fluorescent reporter, providing biologically meaningful estimates of RSV-specific immunity. Finally, the case–cohort design allowed for efficient estimation of associations while maintaining representativeness through appropriate weighting.

### 4.5. Limitations

This study has several limitations. First, our neutralization assay used an RSV-A strain, which may have underestimated the protection against RSV-B. However, cross-protection between RSV types has been reported [44]. Second, although our design enhanced efficiency, subgroup sample sizes were limited, reducing power for some analyses. Third, we were unable to account for postnatal infections with longitudinal changes in antibody levels, as only baseline (cord blood) titers were measured. Fourth, although RSV-neutralizing antibody titers were quantified using a standardized microneutralization assay and expressed as log_2_ IC_50_ values, the results are inherently assay-specific. Consequently, titers cannot be directly compared across studies that differ in viral strains, cell lines, or assay protocols, which may constrain broader interpretation of the data. Fifth, we did not perform molecular characterization of all circulating RSV isolates during the study period. Although partial G gene sequencing was conducted for a subset of hospitalized RSV cases, the antigenic similarity between the assay virus and the overall diversity of locally circulating strains could not be fully determined. If antigenically distinct RSV variants were prevalent, the estimated neutralizing capacity might not fully reflect true protection due to reduced cross-protection.

### 4.6. Public Health Implications

Our findings underscore the potential utility of maternal vaccination in enhancing infant RSV protection, especially when access to monoclonal antibody prophylaxis is limited. The observed associations between lower cord antibody levels and hospitalization risk highlight the need for immunization strategies that ensure sufficient protection during early infancy.

Beyond immunization, improving maternal health may also help protect infants. For instance, addressing maternal anemia—a modifiable and prevalent condition—may contribute to reducing RSV-related hospitalization risk, as suggested by its consistent association in our cohort.

## 5. Conclusions

RSV-ARI hospitalization was common during early infancy in this Vietnamese birth cohort. Lower cord blood RSV-nAb titers were associated with an increased risk of RSV-ARI hospitalization through 12 months of age, with the strongest association within the first six months, whereas maternal anemia showed a consistent association throughout the study period. These results emphasize the importance of maternal health, perinatal immune protection, and support strategies, such as maternal immunization, to prevent severe RSV disease in young infants.

## Figures and Tables

**Table 1:** Characteristics of the subcohort and RSV-ARI hospitalization cases, and hazard ratios for RSV-ARI hospitalization by 24 months of age.

Characteristic	Subcohort (*n* = 392), N (%)	RSV-ARI Hospitalization Cases (*n* = 66), N (%)	Unadjusted HR (95% CI)	Adjusted HR (95% CI)
**Child**				
**Sex**				
Boys	215 (54.9)	43 (65.2)	Reference	Reference
Girls	177 (45.2)	23 (34.9)	0.64 (0.38–1.10)	0.56 (0.32–0.996)
**Birth weight**				
<2500 g	9 (2.3)	0 (0.0)	1.65 × 10^−15^ (8.16 × 10^−16^–3.33 × 10^−15^)	
≥2500 g	383 (97.7)	66 (100.0)	Reference	
**Gestational age**				
<37 weeks	17 (4.3)	4 (6.1)	1.37 (0.46–4.11)	0.94 (0.25–3.45)
≥37 weeks	375 (95.7)	62 (93.9)	Reference	Reference
**Birth season**				
January–March	42 (10.7)	9 (13.6)	Reference	Reference
April–June	93 (23.7)	23 (34.9)	1.15 (0.50–2.67)	1.40 (0.56–3.46)
July–September	172 (43.9)	25 (37.9)	0.64 (0.28–1.47)	0.69 (0.29–1.66)
October–December	85 (21.7)	9 (13.6)	0.46 (0.17–1.23)	0.60 (0.21–1.72)
**RSV antibody titer**				
Low	98 (25.0)	23 (34.9)	1.84 (1.01–3.38)	1.80 (0.93–3.50)
Middle	196 (50.0)	26 (39.4)	Reference	Reference
High	98 (25.0)	17 (25.8)	1.28 (0.67–2.44)	1.32 (0.68–2.58)
**Mother at childbirth**				
**Mode of delivery**				
Vaginal	246 (62.8)	36 (54.6)	Reference	
Cesarean section	146 (37.2)	30 (45.5)	1.43 (0.85–2.41)	
**Maternal age (years)**				
≤24	91 (23.2)	12 (18.2)	Reference	
25–29	144 (36.7)	29 (43.9)	1.49 (0.73–3.04)	
30–34	110 (28.1)	17 (25.8)	1.16 (0.53–2.52)	
≥35	47 (12.0)	8 (12.1)	1.33 (0.51–3.44)	
**Maternal education**				
No school/primary	31 (7.9)	1 (1.5)	0.17 (0.02–1.28)	
Secondary	107 (27.3)	18 (27.3)	0.90 (0.48–1.68)	
High school	90 (23.0)	16 (24.2)	0.92 (0.48–1.76)	
College/university	164 (41.8)	31 (47.0)	Reference	
**Parity**				
Primipara	153 (39.0)	28 (42.4)	1.20 (0.71–2.02)	
Multipara	239 (61.0)	38 (57.6)	Reference	
**Maternal anemia (*n* = 387)**				
Yes	87 (22.5)	35 (53.0)	3.99 (2.35–6.78)	4.10 (2.30–7.28)
No	300 (77.5)	31 (47.0)	Reference	Reference
**Residential area**				
Urban	235 (60.0)	40 (60.6)	0.99 (0.59–1.68)	
Rural	157 (40.1)	26 (39.4)	Reference	

Hazard ratios were estimated using weighted Cox regression models. The adjusted model included antibody titer group (middle as reference), age band (0–5, 6–11, 12–17, and 18–23 months), sex, prematurity, maternal anemia, and birth season. Robust standard errors were clustered by participant ID.

**Table 2:** Characteristics and risk factors of RSV-ARI hospitalization by 12 months of age.

	Subcohort (*n* = 392)	RSV-ARI Hospitalization Cases < 12 m (*n* = 48)	Unadjusted HR (95% CI)	Adjusted HR (95% CI)
**Child**				
**Sex**				
Boys	215 (54.9%)	31 (64.6%)	Reference	Reference
Girls	177 (45.2%)	17 (35.4%)	0.66 (0.36–1.23)	0.57 (0.29–1.12)
**Birth weight**				
<2500 g	9 (2.3%)	0 (0.0%)	1.65 × 10^−15^ (8.04 × 10^−16^–3.38 × 10^−15^)	
≥2500 g	383 (97.7%)	48 (100%)	Reference	
**Gestational age**				
<37 weeks	17 (4.3%)	3 (6.3%)	1.42 (0.41–4.93)	0.81 (0.17–3.85)
≥37 weeks	375 (95.7%)	45 (93.8%)	Reference	Reference
**Birth season**				
January–March	42 (10.7%)	7 (14.6%)	Reference	Reference
April–June	93 (23.7%)	19 (39.6%)	1.21 (0.48–3.07)	1.51 (0.55–4.15)
July–September	172 (43.9%)	17 (35.4%)	0.56 (0.22–1.43)	0.61 (0.23–1.67)
October–December	85 (21.7%)	5 (10.4%)	0.33 (0.10–1.09)	0.47 (0.13–1.66)
**RSV antibody titer**				
Low	98 (25.0%)	20 (41.7%)	2.44 (1.23–4.84)	2.37 (1.09–5.14)
Middle	196 (50.0%)	17 (35.4%)	Reference	Reference
High	98 (25.0%)	11 (22.9%)	1.26 (0.58–2.78)	1.36 (0.61–3.06)
**Mother at childbirth**				
**Mode of delivery**				
Vaginal	246 (62.8%)	24 (50.0%)	Reference	
Cesarean section	146 (37.2%)	24 (50.0%)	1.71 (0.94–3.11)	
**Maternal age (years)**				
≤24	91 (23.2%)	8 (16.7%)	Reference	
25–29	144 (36.7%)	22 (45.8%)	1.69 (0.73–3.94)	
30–34	110 (28.1%)	11 (22.9%)	1.12 (0.44–2.89)	
≥35	47 (12.0%)	7 (14.6%)	1.74 (0.60–5.04)	
**Maternal education**				
No school/primary	31 (7.9%)	1 (2.1)	0.23 (0.03–1.75)	
Secondary	107 (27.3%)	13 (27.1)	0.87 (0.43–1.78)	
High school	90 (23.0%)	11 (22.9)	0.85 (0.40–1.82)	
College/university	164 (41.8%)	23 (47.9)	Reference	
**Parity**				
Primipara	153 (39.0%)	20 (41.7%)	1.15 (0.63–2.10)	
Multipara	239 (61.0%)	28 (58.3%)	Reference	
**Maternal anemia (*n* = 387)**				
Yes	87 (22.5%)	29 (60.4%)	5.35 (2.89–9.92)	5.45 (2.80–10.60)
No	300 (77.5%)	19 (39.6%)	Reference	Reference
**Residential area**				
Urban	235 (60.0%)	30 (62.5%)	1.08 (0.58–1.99)	
Rural	157 (40.1%)	18 (37.5%)	Reference	

Hazard ratios were estimated using weighted Cox regression models restricted to outcomes occurring before 12 months of age. The adjusted model included the antibody titer group (middle as reference), age band (0–5, 6–11 months), sex, prematurity, maternal anemia, and birth season. Robust standard errors were clustered by participant ID.

**Table 3:** Estimated hazard ratios for RSV-ARI hospitalization associated with cord blood antibody titer, based on interaction models with age band (**A**) and maternal anemia (**B**).

**(A) Interaction with age band.**				
Age Band (Months)	Titer Group	aHR	95% CI	*p*-Value
0–5	Low	4.05	1.51–10.89	0.006
	High	1.17	0.33–4.07	0.811
6–11	Low	1.23	0.43–3.58	0.698
	High	1.42	0.52–3.87	0.492
12–17	Low	0.40	0.04–3.54	0.409
	High	2.39	0.71–8.11	0.161
18–23	Low	1.02	0.18–5.72	0.986
	High	7.31 × 10^−10^	2.09 × 10^−10^–2.55 × 10^−9^	<0.001
**(B) Interaction with maternal anemia.**				
Maternal Anemia Status	Titer Group	aHR	95% CI	*p*-Value
No anemia	Low	2.74	1.12–6.69	0.027
	High	1.68	0.65–4.35	0.286
Anemia present	Low	1.25	0.46–3.41	0.662
	High	1.09	0.40–2.96	0.865

(**A**) Hazard ratios were estimated using a weighted Cox regression model including interaction terms between the antibody titer group and age band (0–5, 6–11, 12–17, and 18–23 months). The middle-titer group served as a reference. The model was adjusted for sex, prematurity, maternal anemia, and birth season. Robust standard errors were clustered by participant ID. A significant interaction was observed between titer group and age band (*p* for interaction < 0.001). (**B**) Hazard ratios were estimated using a weighted Cox regression model, including interaction terms between the antibody titer group and maternal anemia. The middle-titer group served as a reference. The model was adjusted for sex, prematurity, age band, and birth season. Robust standard errors were clustered by participant ID. No significant interaction was observed between titer group and maternal anemia (*p* for interaction = 0.53).

**Table 4:** Type-specific adjusted hazard ratios for RSV-A and RSV-B hospitalization by antibody titer group.

Antibody Titer Group	aHR for RSV-A (95% CI)	aHR for RSV-B (95% CI)
Low	2.80 (1.00–7.86) (*n* = 3)	1.76 × 10^10^ (2.02 × 10^9^–1.54 × 10^11^) (*n* = 6)
High	1.46 (0.46–4.63) (*n* = 1)	NE (*n* = 0)

*n* indicates the number of RSV-ARI hospitalization cases in each antibody titer group within the subcohort. NE indicates non-estimable due to separation.

## Data Availability

The de-identified minimal dataset and analysis scripts are in the Supplementary Materials (Files S1–S4). Additional individual-level data are available from the corresponding author on reasonable request, subject to a data-use agreement and, where applicable, ethics approval.

## Supplementary Materials

The following supporting information can be downloaded at: https://www.mdpi.com/article/10.3390/vaccines13090963/s1, Table S1: Sensitivity analysis excluding low-birth-weight infants; Table S2: Comparison of subcohort and full birth cohort characteristics; Table S3: Comparison of RSV-ARI hospitalization cases and non-RSV-ARI hospitalizations in children aged <2 years; File S1: rsvari_foranalysis.dta (de-identified dataset); File S2: rsvari_foranalysis.do (analysis script); File S3: README_minimal.txt; File S4: variable_list_from_dta.csv.

## Abbreviations

The following abbreviations are used in this manuscript:

aHR	Adjusted hazard ratio
IC_50_	50% inhibitory concentration
KHGH	Khanh Hoa General Hospital
LRI	Lower respiratory infection
nAb	Neutralizing antibody
RT-PCR	Reverse transcription polymerase chain reaction
RSV	Respiratory syncytial virus
RSV-ARI	Respiratory syncytial virus-associated acute respiratory infection
