# Supplementary material for: Cord Blood RSV-Neutralizing Antibodies and Risk of Hospitalization for RSV-Associated Acute Respiratory Infection in Vietnamese Children: A Case–Cohort Study
Source: Vaccines (Basel). 2025 Sep 11;13(9):963. doi: 10.3390/vaccines13090963 (PMC12474007; doi:10.3390/vaccines13090963)
Supplement: Supplementary file 1 [file vaccines-13-00963-s001.zip › Supplementary tables_R1.pdf]

Supplementary tables

Supplementary Table S1. Sensitivity analysis excluding low-birth-weight infants

Antibody titer group	Adjusted hazard ratio (aHR)	95% CI	p-value
Low	1.78	0.91–3.47	0.091
High	1.33	0.68–2.60	0.398

Adjusted hazard ratios were derived from a weighted Cox regression model, excluding low-birth-weight infants. The model includes the antibody titer group (middle as reference), age band, sex, prematurity, maternal anemia, and birth season. Robust standard errors were clustered by participant ID.

Supplementary Table S2. Comparison of subcohort and full birth cohort characteristics

Characteristic	Subcohort (n=392), N (%)	Whole cohort (n=1977), N (%)
**Child**		
**Sex**		
Boys	215 (54.9)	1042 (52.7)
Girls	177 (45.2)	935 (47.3)
**Birth weight**		
<2500g	9 (2.3)	24 (1.2)
≥2500g	383 (97.7)	1953 (98.8)
**Gestational age at birth**		
<37 weeks	17 (4.3)	76 (3.8)
≥37 weeks	375 (95.7)	1901 (96.2)
**Birth season**		
Jan–Mar	42 (10.7)	247 (12.5)
Apr–Jun	93 (23.7)	439 (22.2)
Jul–Sep	172 (43.9)	871 (44.1)
Oct–Dec	85 (21.7)	420 (21.2)
**Mother at childbirth**		
**Mode of delivery**		
Vaginal	246 (62.8)	1179 (58.5)
Cesarean section	146 (37.2)	836 (41.5)
**Mother's age (years)**		
≤24	91 (23.2)	434 (22.0)
25–29	144 (36.7)	767 (38.8)
30–34	110 (28.1)	555 (28.1)
≥35	47 (12.0)	221 (11.2)
**Maternal education**		
No school/primary	31 (7.9)	135 (6.8)
Secondary	107 (27.3)	465 (23.5)

Characteristic	Subcohort (n=392), N (%)	Whole cohort (n=1977), N (%)
High school	90 (23.0)	497 (25.1)
College/university	164 (41.8)	880 (44.5)
**Para**		
Primipara	153 (39.0)	815 (41.2)
Multipara	239 (61.0)	1162 (58.8)
**Anemia***		
Yes	87 (22.5)	397 (20.4)
No	300 (77.5)	1553 (79.6)
**Residential area**		
Urban	235 (60.0)	1178 (59.6)
Rural	157 (40.1)	799 (40.4)

* Anemia data available for 387 subcohort members and 1950 full cohort participants.

Supplementary Table S3. Comparison of RSV-ARI hospitalization cases (study cases) and all ARI hospitalizations among children aged <2 years.

Characteristic	RSV-ARI birth cohort (n=66), N (%)	ARI <2y hospitalized from Aug 2018 to Sep 2020 (n=2643), N (%)
**Demographic**		
Sex		
Boys	43 (65.2)	1585 (60.0)
Girls	23 (34.9)	1058 (40.0)
Age (months)		
1–5	23 (34.9)	722 (27.3)
6–11	24 (36.4)	746 (28.2)
12–23	19 (28.8)	1175 (44.5)
**Clinical symptoms**		
Cough	66 (100.0)	2612 (98.8)
Tachypnea	17 (25.8)	720 (27.2)
Chest indrawing	8 (12.1)	465 (17.6)
Stridor	7 (10.6)	457 (17.3)
Wheezing	58 (87.9)	2051 (77.6)
Crackle	19 (28.8)	780 (29.5)
Danger sign*	0 (0.0)	205 (7.8)
Clinical pneumonia**	21 (31.8)	881 (33.3)
**Viruses detected (n=2630)**		
Entero (n=2615)	15 (23.8)	1000 (38.2)
RSV	66 (100.0)	727 (27.6)
FluA	0 (0.0)	186 (7.1)
PIV3	2 (3.0)	102 (3.9)
HAdV (n=2629)	2 (3.3)	94 (3.6)
hMPV (n=2628)	0 (0.0)	79 (3.0)
Boca (n=2629)	2 (3.3)	60 (2.3)
PIV1	0 (0.0)	45 (1.8)
FluB (n=2628)	0 (0.0)	43 (1.6)

Characteristic	RSV-ARI birth cohort (n=66), N (%)	ARI <2y hospitalized from Aug 2018 to Sep 2020 (n=2643), N (%)
HCoV (229E & OC43) (n=2631)	1 (1.5)	32 (1.2)
PIV4	0 (0.0)	17 (0.7)
PIV2	0 (0.0)	14 (0.6)
**Bacteria detected with ≥10^5 CFU/mL (n=2633)**		
S. pneumoniae	17 (25.8)	499 (19.0)
H. influenzae	8 (9.8)	259 (9.8)
M. catarrhalis	6 (9.1)	329 (12.5)
S. aureus	1 (1.5)	96 (3.7)

*Danger signs included any WHO-defined general danger signs (e.g., inability to drink, convulsions, lethargy, or unconsciousness).

**Clinical pneumonia defined based on clinical criteria including cough and/or difficulty breathing plus age-specific respiratory rate or chest indrawing.
